# Increasing Access to Medical Training With Three-Dimensional Printing: Creation of an Endotracheal Intubation Model

**DOI:** 10.2196/12626

**Published:** 2019-04-09

**Authors:** Lily Park, Steven Price-Williams, Alireza Jalali, Kashif Pirzada

**Affiliations:** 1 Faculty of Medicine University of Ottawa Ottawa, ON Canada; 2 Faculty of Science University of Ottawa Ottawa, ON Canada; 3 Department of Innovations in Medical Education Faculty of Medicine University of Ottawa Ottawa, ON Canada; 4 Faculty of Medicine McMaster University Hamilton, ON Canada

**Keywords:** medical education, printing, three-dimensional

## Abstract

**Background:**

Endotracheal intubation (ETI) is a crucial life-saving procedure, where more than 2 failed attempts can lead to further complications or even death. Like all technical skills, ETI requires sufficient practice to perform adequately. Currently, the models used to practice ETI are expensive and, therefore, difficult to access, particularly in the developing world and in settings that lack a dedicated simulation center.

**Objective:**

This study aimed to improve access to ETI training by creating a comparable yet cost-effective simulation model producible by 3-dimensional (3D) printers.

**Methods:**

Open-source mesh files of relevant anatomy from BodyParts3D were modified through the 3D modeling programs Meshlab (ISTI-CNR) and Blender (Blender Foundation). Several prototypes with varying filaments were tried to optimize the ETI simulation.

**Results:**

We have created the novel 3D-printed pediatric ETI model for learners at all levels to practice this airway management skill at negligible costs compared with current simulation models. It is an open-source design available for all medical trainees.

**Conclusions:**

Revolutions in cost and ease of use have allowed home and even desktop 3D printers to become widespread. Therefore, open-source access to the ETI model will improve accessibility to medical training in the hopes of optimizing patient care.

## Introduction

### Background

Endotracheal intubation (ETI) is a major component of advanced airway management. It involves the insertion of a semirigid plastic tube into the trachea to maintain a patient’s airway in cases where this may be compromised or, as a last resort, to administer drugs [1]. It is a crucial life-saving procedure, which, like all technical skills, is subject to a learning curve [2]. It is essential for medical trainees to practice these skills as unsuccessful ETI or more than 2 failed attempts may lead to further health complications and even death [2]. A 2016 systematic review found that students need to perform 1 to 43 ETIs in a clinical setting for a greater than 80% success rate within 2 attempts during elective procedures under optimal conditions [2]. For a greater than 90% success rate within 2 attempts, at least 50 ETIs per student had to be performed [2]. Therefore, to perform successful ETI in nonelective settings, where the incidence of difficult intubation is 20 times higher than in elective settings, medical trainees must exceed 50 ETIs [2].

Although there is a clear suggestion of a positive correlation between practice and successful ETI, accessibility to training is limited. First, many learners will not be able to practice ETI on human subjects until they reach a certain level of competency in their training. To address this barrier, simulation models have been developed and have shown to be comparably effective in training learners. However, these simulation models are expensive and, thus, difficult to access, particularly in developing areas. Priced at an upwards of Can $2000, many training facilities are limited in the purchase and provision of these important learning tools [3].

### Objective

The objective of this study was to design and develop a 3-dimensional (3D)–printed ETI simulation model that can be printed at a low cost. The hope is that improved accessibility to cost-effective training tools will provide more opportunities to practice performing difficult yet important procedures such as ETI.

## Methods

### Design

The foundation for the model design was acquired through BodyParts3D and modified for the needs of the ETI model. As part of an academic project at the Tokyo University Database Center for Life Science, BodyParts3D was built as a repository of free and open-source anatomic models digitized from an actual patient computed tomography (CT) scan [4]. Using clinical images obtained from CT as well as anatomical textbooks and atlases, artists at BodyParts3D have created downloadable 3D model files for disseminated use [4]. Multimedia Appendix 1 outlines the files used to create this model.

The individual .obj files were imported into the 3D modeling software Meshlab and Blender for necessary modifications and manipulation into the human head and neck model. They were exported as .stl files for ease of transfer between workers. The design of facial structures, including the face, eyes, and hair, was outsourced and built upon the foundation of the skull mesh.

**Figure 1:**
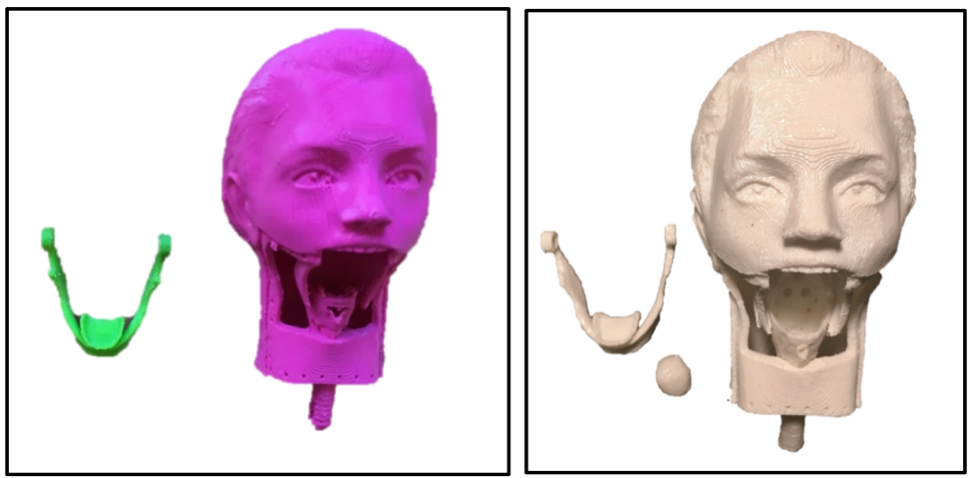
Left: prototype 1 (8.5x6x7.5 cm); right: prototype 2 (11x7.5x8.5 cm).

Overall, 3 prototypes were made for this model. Prototypes 1 and 2 were scaled down to check for the feasibility of the print (Figure 1). Once the prints were satisfactory, prototype 3 was printed as a life-sized pediatric model.

### Material Selection and Print

The head, mandible, throat piece, and tongue were created as separate .stl files for ease of printing and manipulation. Filaments selected to print these models varied depending on intended function and cost. The filaments used throughout this project are compared in Table 1 [5]. Prototypes 1 and 2 were printed through a Makerbot Replicator 2X with double extrusion. These were printed with polylactic acid or acrylonitrile butadiene styrene filaments, and the scaffoldings, designed for support throughout the print, were dissolved with limonene solution. Polyethylene terephthalate glycol modified (PETG) and Ninjaflex were the materials used to print prototype 3. Details on printing prototype 3 will be further discussed.

**Table 1:** Comparing 3-dimensional printing filaments.

Material	Pros	Cons
Polyethylene terephthalate glycol modified	Highest durability, impact resistant, and good layer adhesion—less likely for prints to be warped or to shrink	Requires specific 3-dimensional printing parameters
Polylactic acid	Easily extruded and cost-effective	Less durable
Acrylonitrile butadiene styrene	Most cost-effective, durable, flexible, and easily extruded	Requires higher temperature to reach melting point, more likely for prints to be warped or to shrink, and dangerous fumes during printing
Ninjaflex	High flexibility	Difficult to print

#### Part 1: Head

**Figure 2:**
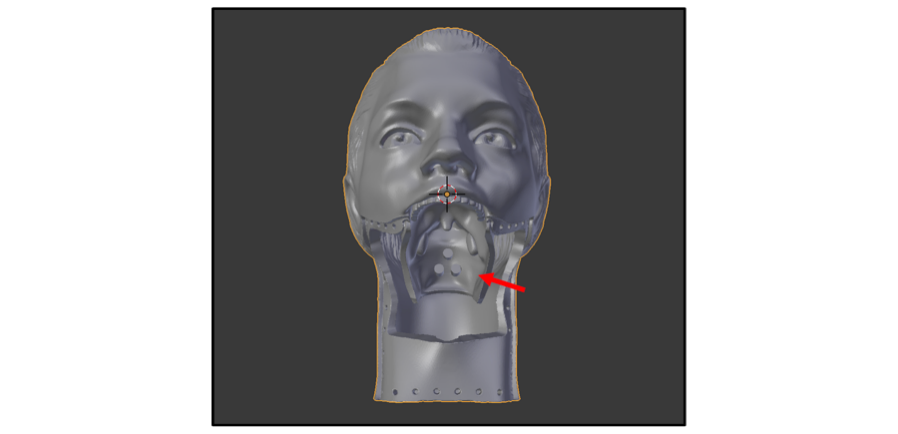
Head piece, unsliced. Red arrow is the base for the soft tissue throat piece.

**Figure 3:**
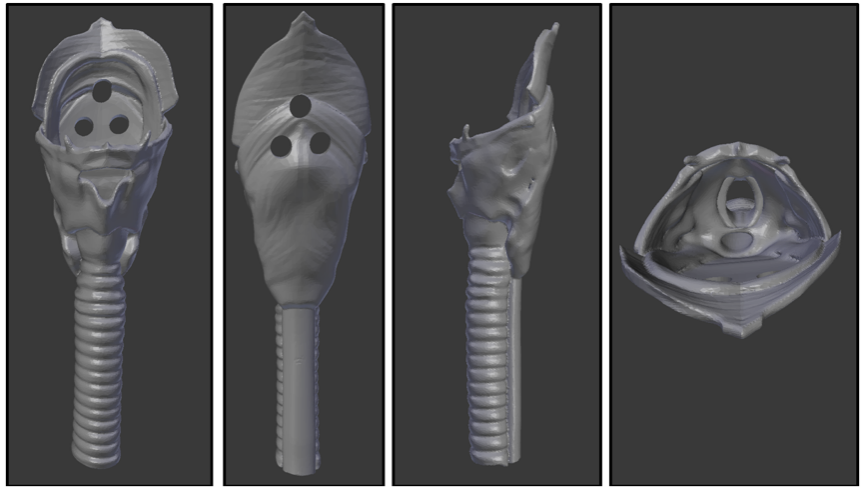
Throat piece. Left to right: anterior view, posterior view, lateral view, cranial-caudal view.

**Figure 4:**
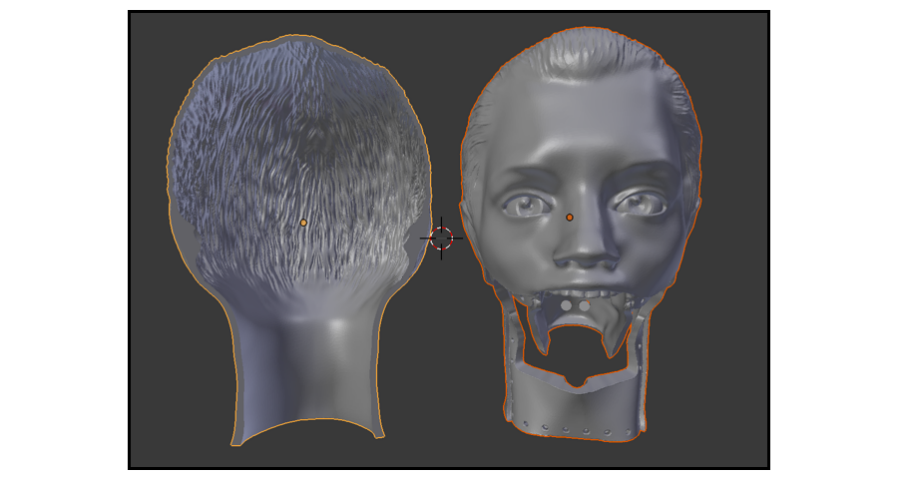
Coronal slices of the head piece, anterior view.

The head is the foundation onto which all the other pieces are assembled (Figure 2). There is a connective piece that continues downward from the maxilla, which provides a base for the throat (Part 2) to attach. Three pegs were created on this connective piece with corresponding holes on Part 2 to facilitate joining of the pieces (Figure 3). The head portion was printed using PETG filaments entirely. Due to the size of the print, the head was sliced along the coronal plane into 2 pieces for ease of printing (Figure 4).

#### Part 2: Throat and Tongue

**Figure 5:**
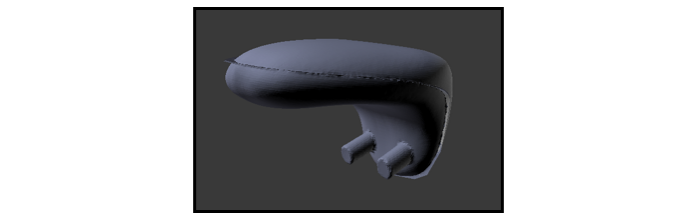
Tongue.

This part includes the trachea, esophagus, and soft tissue, which are pertinent anatomy for learning ETI (Figure 4). The trachea was manually hollowed out to suit the needs of the model. To simulate the soft textures of the throat part, it was printed with Ninjaflex filament, and scaffolding was removed manually. The tongue was also printed with Ninjaflex material and was designed to fit the mandible (Figure 5).

#### Part 3: Mandible

**Figure 6:**
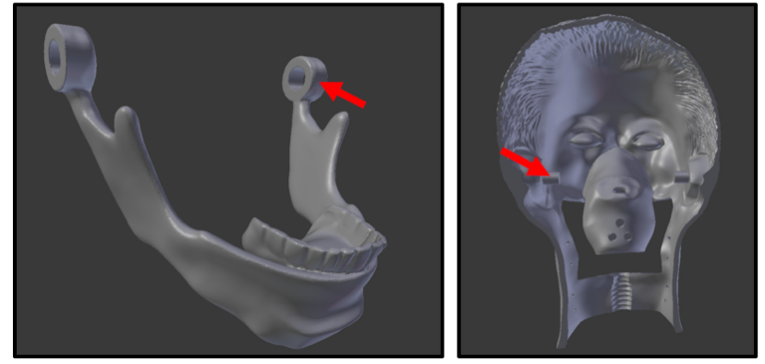
Left: Mandible with hinge. Right: Head piece with corresponding hinge compartment.

The mandible was created separately to allow for articulation with the head piece (Figure 6). There is a hinge component with a corresponding attachment portion embedded on the head piece (Figure 6).

### Assembly

**Figure 7:**
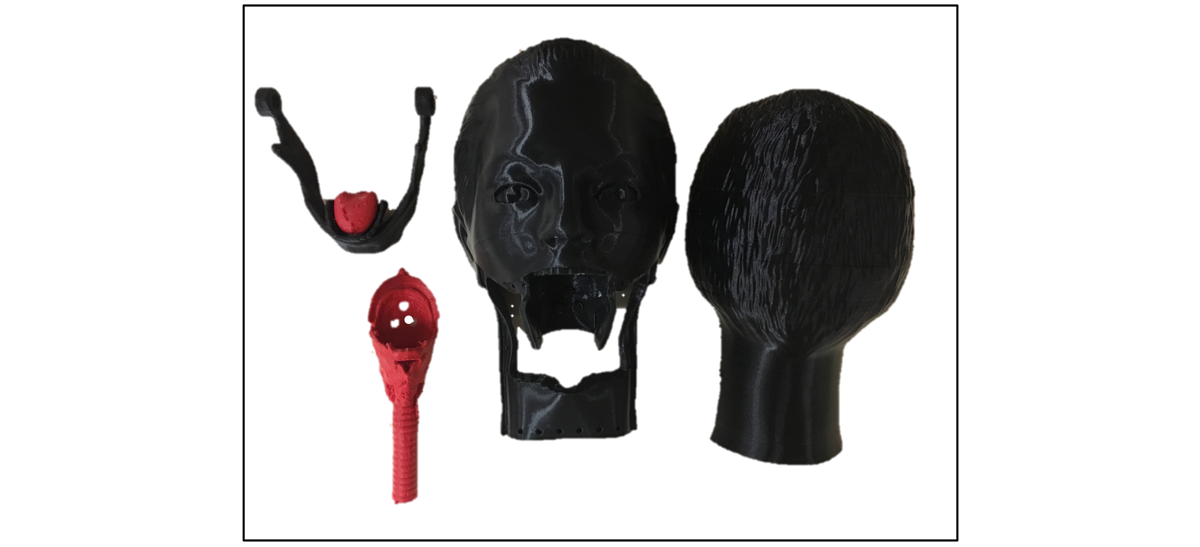
Three-dimensional–printed pieces from left to right: mandible and tongue, throat, head coronal cut 1 and 2. Dimensions: 25.5x16x21 cm.

Upon delivery of the 3D-printed pieces from a third-party vendor, they were assembled in a stepwise fashion (Figure 7). Ethylene-vinyl acetate copolymer was the adhesive of choice. It was used to bind the 2 coronal sections of the head together as well as the throat piece. The mandible was aligned with the corresponding attachment portion on the head and supported with elastic bands to simulate mandibular elevation, depression, and protraction.

## Results

**Figure 8:**
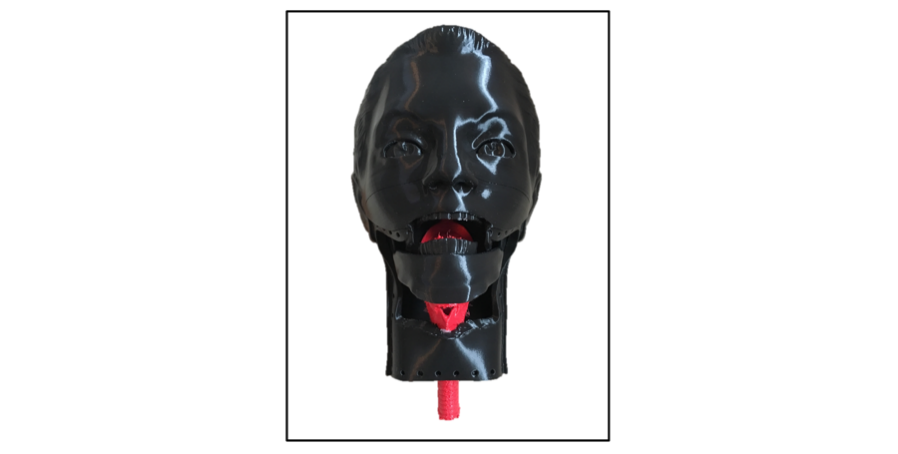
Assembled Endotracheal Intubation Model.

The 3D-printed pieces were combined to create the novel pediatric ETI simulation model (Figure 8). All parts of the model, besides the elastic bands used to support the mandible, were produced by a 3D printer.

### Printing the Model

Prototype 3 was printed by a third-party vendor. The cost to print the entire model from a third-party vendor was approximately Can $130 including taxes and front-door delivery. It was printed through a highly modified Folger Tech FT-5 at-home printer on the following print settings: 6-mm nozzle, 15-mm layers, and 60-mm/s print speed. The entire print took about 60 hours, and around 2.7 lb of generic PETG filament was used. The raw materials used cost around Can $53.90. Assembly took 1 hour and a full day for the adhesive to set.

### Using the Model

The equipment used to practice ETI on this model is as follows: pediatric laryngoscope with miller blades, 5-mm endotracheal tube with stylet, and bag-valve ventilator. A balloon can be attached at the end of the trachea for inflation to signify proper intubation.

## Discussion

### Principal Findings

Limitations on learning opportunities for medical trainees can have impacts that ripple throughout their careers. It is important to improve such opportunities in quality and quantity, especially for training in procedures that require regular practice, such as ETI. Thus, we have explored a new and emerging avenue to increase accessibility to medical training.

Current literature that involves 3D printed models for use in medicine largely surrounds visualization and improved characterization of patient-specific anatomy for teaching or presurgical planning. Efforts to utilize 3D printing for the purposes of medical education, specifically skill building, are few yet emerging. One such example is the 3D-printed trachea for cricothyroidotomy simulation developed by Doucet et al [6]. Like the 3D-printed ETI model, Doucet et al’s trachea is a cost-effective medical simulation model intended to improve training accessibility. Unlike cricothyroidotomy, which is often the last resort in airway management, ETI involves training in head and neck manipulation as well as airway visualization [7]. Therefore, an ETI training simulator requires a full head and neck model in addition to the trachea. Although this increases the complexity of the model as well as the print time and cost, the greater use of ETI compared with cricothyroidotomy in clinical practice merits greater investment in these regards.

### Limitations

There are several technical considerations for reproduction and use of the present ETI model. Of particular note is the significant amount of time required to produce it. In most cases, the print would be ordered through a commercial 3D printing business. However, the size and complexity of the model increase the time required to print, which in turn raises the cost of the model. Furthermore, time must be spent to assemble the parts once they are printed. The model was designed to make assembly as simple as possible. Due to the current technical limitations in 3D printing, the ETI model currently cannot be produced as the intended final product in 1 print. Another consideration is the difference in texture between a 3D-printed object and human anatomy. The PETG filament used for the head and mandible is hard plastic and does not resemble human skin. This is not concerning as the external skin is not a significant anatomical feature associated with performing ETI. However, the throat and tongue present as limitations. Ninjaflex was used to print these parts as it is the filament most comparable with soft tissue that is commercially available. Still, it is slightly unyielding compared with human anatomy, which impacts the accuracy of the simulation.

### Future Directions

With rapid advancements in 3D printing and modeling technologies, there is ever-growing potential to enhance this model. Introduction of new filaments and rapid improvements of 3D printers may soon resolve the aforementioned technical considerations. Furthermore, certain aspects of the model can be modified to suit the needs of the learner. For example, to simulate particularly challenging airways, several premade tongue and mandible prints of varying sizes and abnormalities can be made available.

Future steps for the 3D-printed ETI model include model validation by demonstrating noninferiority compared with current commercial ETI simulation models. Trainees of all levels can be stratified to perform ETI in both the 3D-printed and commercial simulation models. A questionnaire may then be distributed to identify differences in ETI training between the 2 models. If areas for improvement are identified to optimize the model, these adjustments may accordingly be made to the open-source design for the user’s needs.

### Conclusions

The 3D-printed ETI model is a significantly cost-effective option for trainees to practice ETI compared to its current commercial counterparts. With the aforementioned instruments, ETI can be successfully performed with impressive comparability to the current simulation models. Therefore, as an open-source design, our model has the potential to increase accessibility for medical trainees to practice this challenging and important procedure. By improving training accessibility, the ETI model is the realized potential of 3D printing’s impact on medicine. It stands as a precedent for future models that will similarly aim to improve clinical practice by addressing medical education needs for future health care providers.

Access to the open-source model is available on the Github website [8].

## Appendix

Multimedia Appendix 1 Files obtained from BodyParts3D.

## Abbreviations

3D: 3-dimensional
CT: computed tomography
ETI: endotracheal intubation
PETG: polyethylene terephthalate glycol modified
