# Supplementary material for: Increasing Access to Medical Training With Three-Dimensional Printing: Creation of an Endotracheal Intubation Model
Source: JMIR Med Educ. 2019 Apr 9;5(1):e12626. doi: 10.2196/12626 (PMC6477567; doi:10.2196/12626)
Supplement: Multimedia Appendix 1 [file mededu_v5i1e12626_app1.pdf]

Multimedia Appendix

Head Piece	FJ19_BP1741_FMA52788_right parietal Bone<br>FJ20_BP694_FMA5364_Right maxilla<br>FJ56_BP702_FMA53655_right palatine bone<br>FJ86_BP1736_FMA52738_Right temporal bone<br>FJ119_BP699_FMA52736_Sphenoid bone<br>FJ120_BP658_FMA53647_Right nasal bone<br>FJ172_BP703_FMA53656_Left palatine bone<br>FJ214_BP1763_FMA52893_Left zygomatic bone<br>FJ419_BP659_FMA53648_left nasal bone<br>FJ531_BP715_FMA54738_Left inferior nasal concha<br>FJ580_BP698_FMA52739_Left temporal bone<br>FJ607_BP649_FMA52734_Frontal bone<br>FJ640_BP1738_FMA52740_ethmoid<br>FJ721_BP705_FMA52892_Right zygomatic bone<br>FJ803_BP1740_FMA52735_occipital bone<br>FJ806_BP1751_FMA9710_Vomer<br>FJ818_BP1748_FMA54737_Right inferior nasal concha<br>FJ821_BP1482_FMA71704_Set of nasal cartilages<br>FJ6468_BP21998_FMA53649_Right maxilla<br>FJ6380_BP22020_FMA53650_Left maxilla<br>FJ57_BP950_FMA55689_Right upper first secondary premolar tooth<br>FJ149_BP917_FMA55783_Left upper lateral secondary incisor tooth<br>FJ191_BP936_FMA55699_Left upper first secondary molar tooth<br>FJ347_BP966_FMA55799_Left upper secondary canine tooth<br>FJ388_BP965_FMA55798_Right upper secondary canine tooth<br>FJ451_BP916_FMA55680_Right upper lateral secondary incisor tooth<br>FJ452_BP932_FMA55697_Right upper second secondary molar tooth<br>FJ480_BP947_FMA55688_Right upper second secondary premolar tooth<br>FJ490_BP948_FMA55691_Left upper second secondary premolar tooth<br>FJ513_BP920_FMA55682_Left upper central secondary incisor tooth<br>FJ561_BP919_FMA55681_Right upper central secondary incisor tooth<br>FJ641_BP951_FMA55690_Left upper first secondary premolar tooth<br>FJ773_BP935_FMA55698_Right upper first secondary molar tooth<br>FJ774_BP933_FMA55700_Left upper second secondary molar tooth

Throat piece	FJ346_BP1765_FMA52749_Hyoid bone<br>FJ675_BP2282_FMA55099_Thyroid cartilage<br>cricoid<br>FJ523_BP2540_FMA13352_Right thyrohyoid<br>FJ743_BP2541_FMA13353_Left thyrohyoid<br>FJ519_BP3451_FMA7394_Trachea<br>FJ854_BP4618_FMA7131_Esophagus<br>FJ2740_BP20100_FMA46636_Left inferior pharyngeal constrictor<br>FJ2742_BP23923_FMA46633_Right middle pharyngeal constrictor<br>FJ2752_BP23848_FMA46635_Right inferior pharyngeal constrictor<br>FJ2754_BP23937_FMA46634_Left middle pharyngeal constrictor<br>FJ2755_BP23433_FMA46671_Right palatopharyngeus<br>FJ2743_BP23494_FMA46672_Left palatopharyngeus<br>FJ2747_BP20320_FMA46632_Left superior pharyngeal constrictor<br>FJ2759_BP20310_FMA46631_Right superior pharyngeal constrictor
Mandible	FJ150_BP955_FMA55693_Left lower first secondary premolar tooth<br>FJ151_BP939_FMA55706_Right lower secondary molar tooth<br>FJ173_BP927_FMA57143_Left lower central secondary incisor tooth<br>FJ216_BP943_FMA55704_Left lower first secondary molar tooth<br>FJ243_BP954_FMA55694_Right lower first secondary premolar tooth<br>FJ321_BP924_FMA57141_Left lower lateral secondary incisor tooth<br>FJ387_BP957_FMA55695_Right lower second secondary premolar tooth<br>FJ453_BP926_FMA57142_Right lower central secondary incisor tooth<br>FJ479_BP962_FMA55686_Right lower secondary canine tooth<br>FJ481_BP940_FMA55703_Left lower second secondary molar tooth<br>FJ542_BP958_FMA55692_Left lower second secondary premolar tooth<br>FJ676_BP923_FMA57140_Right lower lateral secondary incisor tooth<br>FJ723_BP963_FMA55687_Left lower secondary canine tooth<br>FJ865_BP942_FMA55705_Right lower first secondary molar tooth<br>FJ699_BP709_FMA52748_Mandible
Tongue	FJ2761_BP22597_FMA54640_Tongue
